# Associations between the signing status of family doctor contract services and cervical cancer screening behaviors: a cross-sectional study in Shenzhen, China

**DOI:** 10.1186/s12889-023-15462-9

**Published:** 2023-03-28

**Authors:** Wei Lin, Weikang Huang, Chaofan Mei, Peiyi Liu, He Wang, Shixin Yuan, Xiaoshan Zhao, Yueyun Wang

**Affiliations:** 1 Department of Healthcare, Affiliated Shenzhen Maternity and Child Healthcare Hospital, Southern Medical University, Shenzhen, 518048 China; 2 Research Team of Cervical Cancer Prevention Project in Shenzhen, Affiliated Shenzhen Maternity and Child Healthcare Hospital, Southern Medical University, Shenzhen, 518028 China; 3 Research Institute of Maternity and Child Medicine, Affiliated Shenzhen Maternity and Child Healthcare Hospital, Southern Medical University, Shenzhen, 518028 China

**Keywords:** Family doctor contract services, Cervical cancer, Past screening participation, Future screening willingness

## Abstract

**Background:**

As a core part of the primary healthcare system, family doctor contract services (FDCS) may help healthcare providers promote cervical cancer screening to the female population. However, evidence from population-based studies remains scant. This study aimed to investigate the potential associations between the signing status of FDCS and cervical cancer screening practices in Shenzhen, China.

**Methods:**

A cross-sectional survey among female residents was conducted between July to December 2020 in Shenzhen, China. A multistage sampling method was applied to recruit women seeking health services in community health service centers. Binary logistic regression models were established to assess the associations between the signing status of FDCS and cervical cancer screening behaviors.

**Results:**

Overall, 4389 women were recruited (mean age: 34.28, standard deviation: 7.61). More than half (54.3%) of the participants had signed up with family doctors. Women who had signed up for FDCS performed better in HPV-related knowledge (high-level rate: 49.0% vs. 35.6%, P<0.001), past screening participation (48.4% vs. 38.8%, P<0.001), and future screening willingness (95.9% vs. 90.8%, P<0.001) than non-signing women. Signing up with family doctors was marginally associated with past screening participation (OR: 1.13, 95%CI: 0.99–1.28), which tended to be robust among women with health insurance, being older than 25 years old at sexual debut, using condom consistently during sexual intercourse, and with a low level of HPV related knowledge. Similarly, signing up with family doctors was positively associated with future screening willingness (OR: 1.68, 95%CI: 1.29–2.20), which was more pronounced among women who got married and had health insurance.

**Conclusions:**

This study suggests that signing up with family doctors has positive associations with cervical cancer screening behaviors among Chinese women. Expanding public awareness of cervical cancer prevention and FDCS may be a feasible way to achieve the goal of cervical cancer screening coverage.

## Introduction

Cervical cancer is the fourth most frequent malignancy in females, leading to a heavy burden of 570,000 cases and 311,000 deaths worldwide [1]. Over 99% of cervical cancer cases are attributed to the infection of high-risk human papillomavirus (HPV) [2]. Except for vaccination against HPV infections, early detection of precancers in the cervix via routine screening is regarded as the most effective way to eliminate cervical cancer. Evidence from the UK has shown that primary healthcare plays a key role in preventing cervical cancer as the health providers will discuss cancer risk with the patients and promote risk reduction strategies [3]. In the primary healthcare system of China, community health service centers act as vital frontline places in the network of cervical cancer prevention and control [4]. A community health service center usually provides basic medical care services related to cervical cancer prevention in Shenzhen city [5], such as health consultation, HPV vaccination, gynecological examination, cervical sampling, interpretation of screening results, referral to hospitals for abnormal results, etc. Despite the accessibility of screening services, the key to promoting screening participation lies in increasing the knowledge of cervical cancer prevention and the awareness of screening services among the female population as previous studies have indicated [6], in which frontline health workers usually undertake the responsibility to disseminate correct health information and guide proper screening behaviors [7]. Here, a contract for family doctor services may be a useful tool that greatly helps to build the connection between healthcare providers and the female population in fighting against cervical cancer.

Family doctor contract services (FDCS) have been promoted in mainland China since 2009 as a result of medical reform [8]. It takes a “gate-keeper” role in the hierarchical medical system, providing comprehensive and continuing community health care [9]. In Shenzhen city, residents can choose to sign up with a preferred family doctor team that consists of general practitioners (as the team leader), nurses, and public health doctors from community health service centers nearby [10]. As the contract generally lasts for at least one year, family doctors will keep a relatively stable relationship with the signing resident. Under the working principles, family doctors will offer standardized services, e.g. the establishment and maintenance of health records, delivery of basic medical care and public health services, personalized health support if required (home visits or home care beds), etc. [10]. In this context, people who have signed up for FDCS may be more likely to capture the up-to-date message and engage in health management behaviors, as researchers have found among noncommunicable disease patients [11]. Previous studies have found that visiting general practitioners may help to enhance the uptake of cervical cancer screening in European countries [12, 13], which indicates the potential role of family doctors in facilitating cervical cancer screening at the community level. However, further evidence from population-based studies still remains scant.

Given these research gaps, this study aimed to investigate the utilization of both FDCS and cervical cancer screening services among female residents based on a cross-sectional survey in Shenzhen city. Furthermore, whether signing up with family doctors impact women’s behaviors on cervical cancer screening was also evaluated with particular interest.

## Methods

### Study design and sampling

**Fig. 1:**
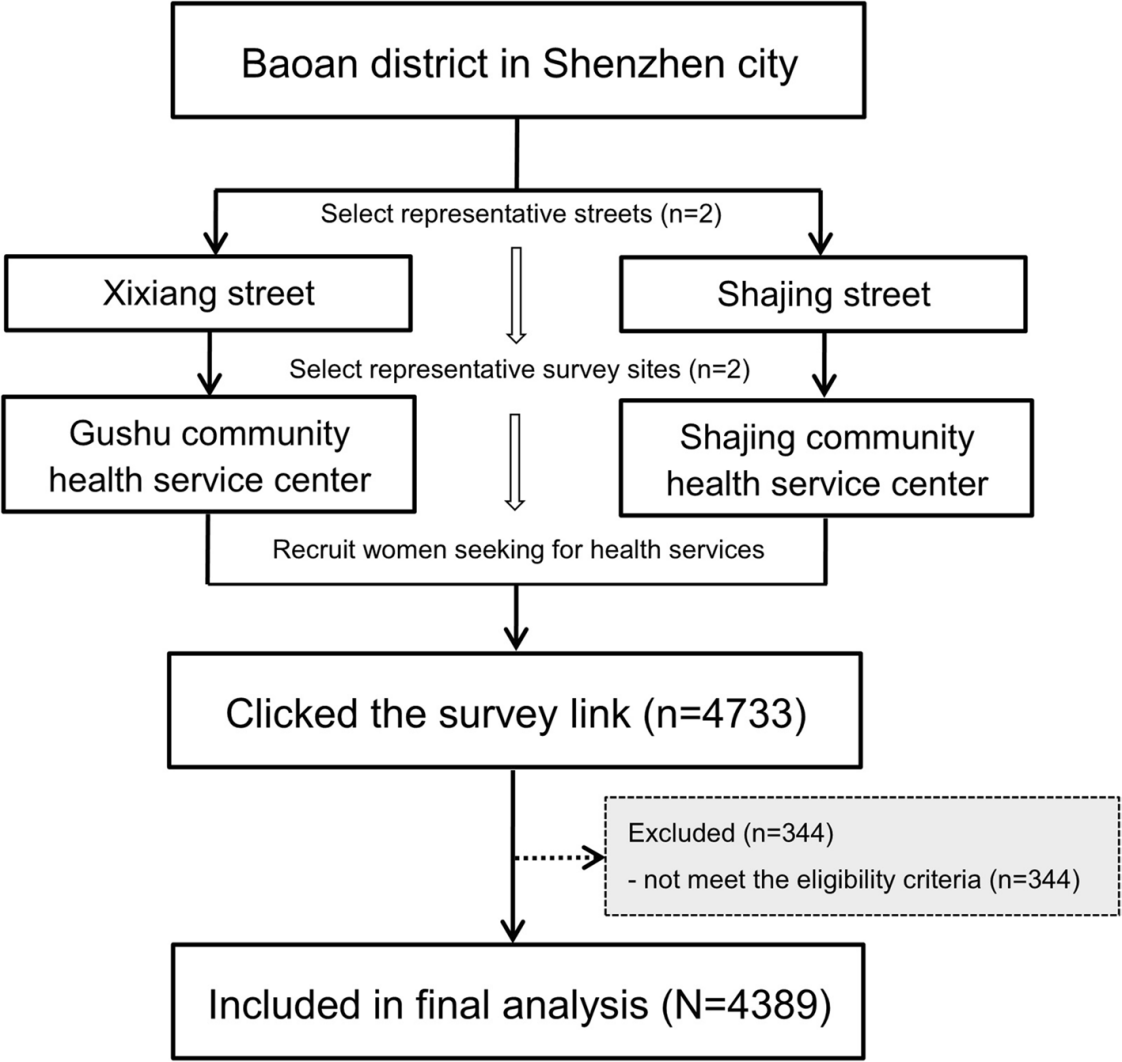
Flow chart diagram of the study sampling

This community-based cross-sectional study was conducted in the Baoan district of Shenzhen city, China between July to December 2020. According to the latest data of the seventh national census, more than 4.47 million residents are living in the Baoan district, which ranks as the most populous district in Shenzhen. A multistage sampling method was applied to recruit participants (Fig. 1). First, two representative administrative streets (Xixiang Street and Shajing Street) in the Baoan district were selected by simple random sampling. Subsequently, one regional community health service center was randomly chosen to be the survey site on these two streets, respectively. During the survey period, women who received health services in the survey sites would be invited to participate in this survey. Eligible participants were defined to be aged from 21 to 65 years, living in the responding district, engaged in sexual intercourse, and willing to participate in the survey. With the help of medical staff, candidate women were provided with a full introduction of the study objectives, contents, and procedures, followed by a Quick Response code linking to the electronic questionnaire. Through scanning the two-dimensional barcode with their smartphones, women were asked to confirm their voluntary participation for informed consent, and then got access to the questionnaire. The questionnaire was hosted by a popular Chinese survey platform named WenJuanXing (Changsha Haoxing Information Technology Co., Ltd., China).

The sample quantity was calculated using the formula of the cross-sectional study: n = μ^2^_α_p(1–p)/δ^2^. Here, α = 0.05 (two sides), μ_α_ = 1.96, δ = 0.015, and the screening rate of cervical cancer in Shenzhen *p* = 35.1% (according to previous findings). The required sample size was 3890. With the no-response rate controlled within 10%, the final sample size was determined to be 4280. During the survey period, a total of 4733 women clicked the online link of this survey. We further excluded 344 respondents who did not meet the eligibility criteria, leaving 4389 participants in the final database.

### Data collection

The present questionnaire mainly focused on evaluating knowledge related to HPV, awareness, participation, and willingness of cervical cancer screening, accompanied by information on socio-demographic characteristics, sexual behavioral characteristics, pre-existing health conditions, health services utilization, etc.

In this survey, whether women were receiving the family doctor contract service was measured by asking ‘Have you already signed up with family doctors in Shenzhen?’ (Yes/No). Otherwise, women needed to offer the major reason for not signing. Women were required to estimate HPV-related knowledge with a total of nine-question items (Cronbach’s alpha in this sample: 0.91) if they had heard of HPV before. For each question, three answer options (Yes/No/Don’t know) were provided. Here, the answer of ‘Don’t know’ was considered to be incorrect. A sum score of nine items (range: 0–9) was assigned according to the number of correct answers. Women who had never heard of HPV or scored below 6 were regarded as with a low level of HPV-related knowledge. Awareness of cervical cancer screening was estimated by asking ‘Have you ever heard of cervical cancer screening?’. If a positive answer was provided, women further needed to answer which screening method they know. Past screening participation was assessed by asking ‘Have you ever participated in cervical cancer screening? (Yes/No)’. If a woman had ever received screening services, details of the latest screening were collected, such as the screening time, method, and result. Willingness of cervical cancer screening was measured by the question ‘Are you willing to receive cervical cancer screening in the following days? (Yes/No)’. Furthermore, women were required to choose the expected screening cost and frequency if they were ready to be screened.

### Statistical analysis

All variables were categorically presented with frequency and percentage (%). The Chi-square test was applied to detect distributed differences in HPV-related knowledge, cervical cancer screening related awareness, and behaviors between the signing and non-signing groups. Binary logistic regression models were established to assess the associations between signing up with family doctors and cervical cancer screening behaviors. We established three models in the following order: model 1 (unadjusted), model 2 (adjusted for age, ethnicity, local household registration, marital status, education level, occupation type, monthly income, and health insurance), and model 3 (further adjusted for age at sexual debut, the number of lifetime sexual partners, consistent condom use during sexual intercourse, parity, and HPV related knowledge based on model 2). The odds ratios (OR) and 95% confidence intervals (CI) were calculated. In addition, stratified analyses were further conducted according to all adjusted variables to detect potential modified effects, in which multiplicative interactions between modified factors and the signing status were calculated by including the product terms in multivariate logistic regression models. All statistical analyses were performed by using SPSS software (version 21.0, IBM SPSS Statistics, New York, United States). The statistical significance level was set to be *P* < 0.05 (two-sided).

## Results

A total of 4389 participants were included in the final analysis (mean age: 34.28, standard deviation: 7.61). More than half (54.3%) of them had signed up with family doctors. The most common reason for not signing was that women did not know FDCS before (35.8%). Characteristics of the study subjects were shown in Table 1.

**Table 1:** Characteristics of all participants (*N* = 4389)

Variable	Number	Frequency (%)
**Age (year)**		
21–30	1505	34.3
31–40	2013	45.9
41–65	871	19.8
**Ethnicity**		
Han	4034	91.9
Others	355	8.1
**Local household registration**		
Yes	568	12.9
No	3821	87.1
**Marital status**		
Single/divorced/widow	547	12.5
Married	3842	87.5
**Education level**		
Junior middle school or below	1853	42.2
Senior middle school	1239	28.2
College or above	1297	29.6
**Occupation type**		
Administrators/Professionals	780	17.8
Workers	1460	33.3
Business services personnel	671	15.3
Housewife/ unemployed women	792	18.0
Others	686	15.6
**Monthly income (RMB)**		
< 5,000	2470	56.3
5,000–7999	1288	29.3
≥ 8000	631	14.4
**Health insurance**		
No	527	12.0
Yes	3862	88.0
**Age at sexual debut (year)**		
< 18	470	10.7
18–24	3104	70.7
≥ 25	815	18.6
**The number of lifetime sexual partners**		
1	3191	72.7
2	743	16.9
≥ 3	455	10.4
**Consistent condom use during sexual intercourse**		
No	3585	81.7
Yes	804	18.3
**Parity**		
0	600	13.7
1	1390	31.7
2	1937	44.1
≥ 3	462	10.5
**Signing up with family doctors**		
No	2004	45.7
Yes	2385	54.3
**The main reason for not signing up with family doctors** ^a^		
Never heard of FDCS	717	35.8
Fear of hassles	145	7.2
Thinking it useless	84	4.2
Unknowing how to sign up with family doctors	459	22.9
Other reasons	599	29.9

^a^Women who had signed up with family doctors were not required to answer this question

Among the participants, 62.6% of them had heard of HPV, but only 42.9% of them had a high level of HPV-related knowledge. Regarding the HPV knowledge question items, women signing up with family doctors demonstrated significantly higher correct rates of 8 question items than non-contracted counterparts (all P<0.05), except for Q1 (*P* = 0.72). Distinct levels of HPV-related knowledge existed between the signing and non-signing groups (high-level rate: 49.0% vs. 35.6%, P<0.001) (Table 2). Most of the respondents (84.4%) had heard of cervical cancer screening, but only 44.0% of them had been screened before. Moreover, 93.6% of them were willing to be screened in the following days. Compared to non-signing women, women signing up with family doctors performed better in awareness (89.9% vs. 77.8%, P<0.001), past screening participation (48.4% vs. 38.8%, P<0.001), and future screening willingness (95.9% vs. 90.8%, P<0.001) of cervical cancer screening (Table 3). Moreover, signing women were more likely to hear of specific screening methods, such as HPV testing, cytology, and visual inspection methods (all P<0.05). Signing women also tended to be screened within 3 years, to have fewer unknown screening methods, and to expect free and frequent screening services (all P<0.05).

**Table 2:** HPV-related knowledge varied by the signing status of family doctor (*N* = 4389)

Variable	Signing up with family doctors		P for chi square test
	**No, n(%)**	**Yes, n(%)**	
**Heard of HPV**			
No	878 (43.8)	763 (32.0)	** < 0.001**
Yes	1126 (56.2)	1622 (68.0)	
**HPV knowledge question item** ^a^			
**Q1. HPV is very rare**			
Correct	534 (47.4)	758 (46.7)	0.72
Wrong/unknown	592 (52.6)	864 (53.3)	
**Q2. HPV infection is mainly transmitted through sexual contact**			
Correct	770 (68.4)	1246 (76.8)	** < 0.001**
Wrong/unknown	356 (31.6)	376 (23.2)	
**Q3. HPV can cause cervical cancer**			
Correct	869 (77.2)	1363 (84.0)	** < 0.001**
Wrong/unknown	257 (22.8)	259 (16.0)	
**Q4. HPV can cause genital warts**			
Correct	750 (66.6)	1187 (73.2)	** < 0.001**
Wrong/unknown	376 (33.4)	435 (26.8)	
**Q5. People with an earlier age at sex debut are susceptible to HPV acquisition**			
Correct	644 (57.2)	1084 (66.8)	** < 0.001**
Wrong/unknown	482 (42.8)	538 (33.2)	
**Q6. Most sexually active people will get HPV at some point in their lives**			
Correct	922 (81.9)	1420 (87.5)	** < 0.001**
Wrong/unknown	204 (18.1)	202 (12.5)	
**Q7. Having multiple sexual partners may increase the risk of getting HPV infection**			
Correct	899 (79.8)	1405 (86.6)	** < 0.001**
Wrong/unknown	227 (20.2)	217 (13.4)	
**Q8. Using Condom may reduce the chance of HPV transmission**			
Correct	599 (53.2)	991 (61.1)	** < 0.001**
Wrong/unknown	527 (46.8)	631 (38.9)	
**Q9. Most HPV infections can be cleared by human autoimmunity**			
Correct	482 (42.8)	838 (51.7)	** < 0.001**
Wrong/unknown	644 (57.2)	784 (48.3)	
**HPV-related knowledge** ^b^			
Low level (< 6)	1290 (64.4)	1217 (51.0)	** < 0.001**
High level (≥ 6)	714 (35.6)	1168 (49.0)	

^a^Women who had never heard of HPV were not required to answer HPV knowledge questions

^b^Women who had never heard of HPV were regarded with a low level of HPV-related knowledge

**Table 3:** Awareness, experience, and attitudes towards cervical cancer screening varied by the signing status of family doctor (*N* = 4389)

Variable	Signing up with family doctors		P for chi square test
	**No, n(%)**	**Yes, n(%)**	
**Heard of cervical cancer screening**			
No	444 (22.2)	241 (10.1)	** < 0.001**
Yes	1560 (77.8)	2144 (89.9)	
**Heard of HPV testing methods** ^a^			
No	751 (48.1)	876 (40.9)	** < 0.001**
Yes	809 (51.9)	1268 (59.1)	
**Heard of cytology methods** ^a^			
No	976 (62.6)	1090 (50.8)	** < 0.001**
Yes	584 (37.4)	1054 (49.2)	
**Heard of visual inspection methods** ^a^			
No	1378 (88.3)	1819 (84.8)	**0.002**
Yes	182 (11.7)	325 (15.2)	
**Past screening participation**			
No	1226 (61.2)	1231 (51.6)	** < 0.001**
Yes	778 (38.8)	1154 (48.4)	
**The time of last screening** ^b^			
Within 3 years	643 (82.6)	1009 (87.4)	**0.003**
Over 3 years	135 (17.4)	145 (12.6)	
**The screening method of last screening** ^b^			
HPV testing	171 (22.0)	291 (25.2)	**0.001**
Cytology	62 (8.0)	87 (7.5)	
Co-testing	204 (26.2)	369 (32.0)	
Unknown	341 (43.8)	407 (35.3)	
**The result of last screening** ^b^			
Normal	582 (74.8)	916 (79.4)	0.062
Abnormal	139 (17.9)	168 (14.6)	
Unknown	57 (7.3)	70 (6.1)	
**Future screening willingness**			
No	185 (9.2)	97 (4.1)	** < 0.001**
Yes	1819 (90.8)	2288 (95.9)	
**Expected screening cost (CNY)** ^c^			
None	679 (37.3)	1052 (46.0)	** < 0.001**
≤ 100	257 (14.1)	312 (13.6)	
101–200	339 (18.6)	350 (15.3)	
201–300	242 (13.3)	263 (11.5)	
≥ 301	302 (16.6)	311 (13.6)	
**Expected screening frequency** ^c^			
Every year	1252 (68.8)	1722 (75.3)	** < 0.001**
Every three year	258 (14.2)	315 (13.8)	
Every five year	13 (0.7)	11 (0.5)	
Depends on the doctor’s suggestions	246 (13.5)	220 (9.6)	
Other expectations	50 (2.7)	20 (0.9)	

^a^Women who had never heard of cervical cancer screening were not required to answer these questions

^b^Women who had never been screened were not required to answer these questions

^c^Women who were not willing to screen were not required to answer these questions

**Fig. 2:**
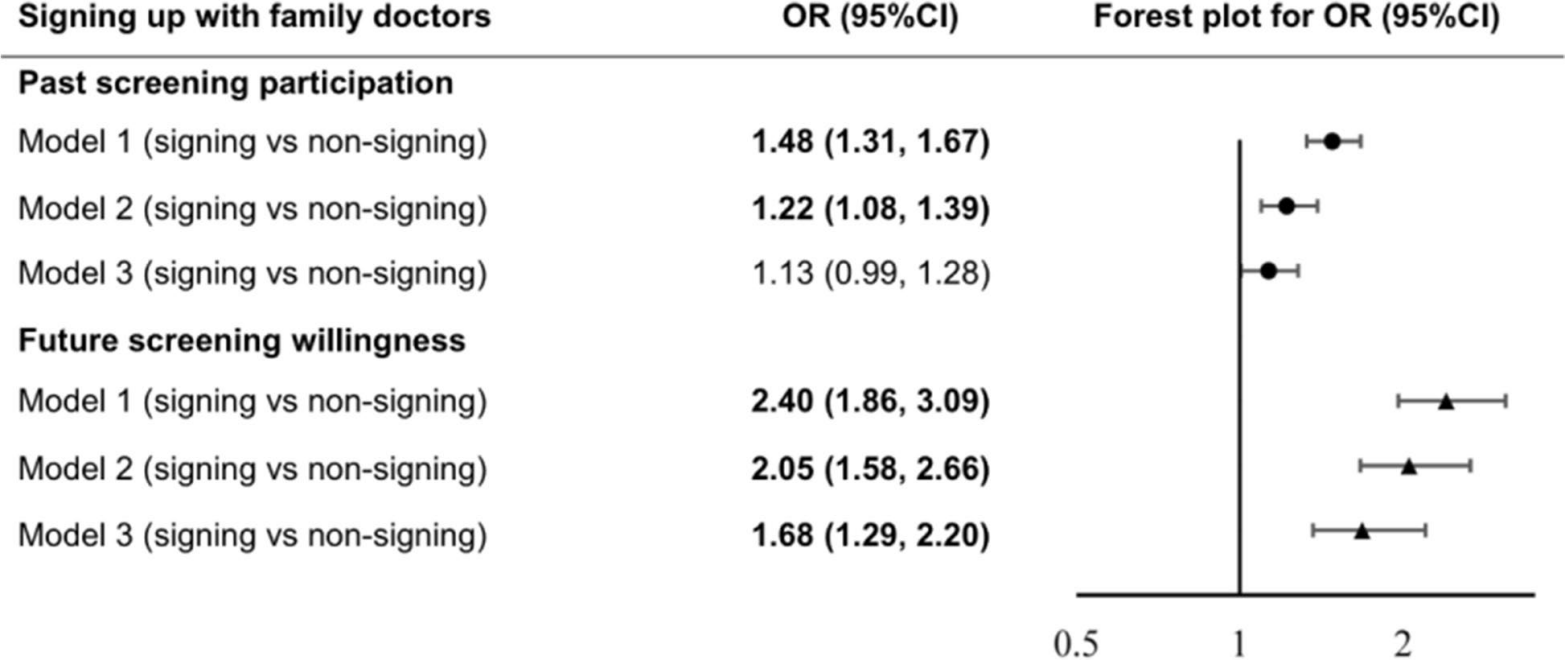
Associations between signing up with family doctors and cervical cancer screening behaviors

After adjusting for potential confounding variables, signing up with family doctors was marginally associated with past screening participation (OR: 1.13, 95%CI: 0.99–1.28) when compared to their non-signing counterparts (Fig. 2). When stratified by socio-demographic characteristics, the effect of FDCS on past screening participation tended to be robust among women with health insurance (OR: 1.21, 95%CI: 1.05–1.39), being older than 25 years old at sexual debut (OR: 1.59, 95%CI: 1.16–2.18), using condom consistently during sexual intercourse (OR: 1.55, 95%CI: 1.11–2.16), and with a low level of HPV related knowledge (OR: 1.39, 95%CI: 1.17–1.65). Multiplicative interactions of the signing status with health insurance, age at sexual debut, condom use, and HPV-related knowledge level were detected (P for multiplicative interactions: <0.001, 0.009, 0.042, and 0.004, respectively) (Table 4).

**Table 4:** Modified effects of socio-demographic, sexual behavioural factors and HPV-related knowledge on the associations between signing up with family doctors and cervical cancer screening behaviors (*N* = 4389)

Modified factor	Signing up with family doctors	Past screening participation		Future screening willingness	
		**OR (95%CI)** ^a^	**OR (95%CI)** ^b^	**OR (95%CI)** ^a^	**OR (95%CI)** ^b^
**Age (year)**					
21–30	No	1.00 (reference)	1.00 (reference)	1.00 (reference)	1.00 (reference)
	Yes	**1.53 (1.22, 1.92)**	1.24 (0.97, 1.59)	**2.35 (1.46, 3.55)**	**1.73 (1.11, 2.72)**
31–40	No	1.00 (reference)	1.00 (reference)	1.00 (reference)	1.00 (reference)
	Yes	**1.36 (1.14, 1.62)**	1.11 (0.92, 1.34)	**2.15 (1.44, 3.21)**	**1.58 (1.04, 2.41)**
41–65	No	1.00 (reference)	1.00 (reference)	1.00 (reference)	1.00 (reference)
	Yes	1.18 (0.90, 1.55)	1.17 (0.86, 1.60)	**2.84 (1.64, 4.91)**	**2.29 (1.23, 4.29)**
P for multiplicative interaction		0.36	0.67	0.72	0.71
**Ethnicity**					
Han	No	1.00 (reference)	1.00 (reference)	1.00 (reference)	1.00 (reference)
	Yes	**1.46 (1.29, 1.66)**	1.10 (0.96, 1.26)	**2.46 (1.88, 3.21)**	**1.73 (1.30, 2.30)**
Others	No	1.00 (reference)	1.00 (reference)	1.00 (reference)	1.00 (reference)
	Yes	**1.60 (1.04, 2.44)**	1.42 (0.86, 2.33)	1.81 (0.82, 4.01)	1.59 (0.59, 4.31)
P for multiplicative interaction		0.70	0.21	0.47	0.46
**Local household registration**					
Yes	No	1.00 (reference)	1.00 (reference)	1.00 (reference)	1.00 (reference)
	Yes	1.06 (0.74, 1.51)	0.83 (0.55, 1.25)	1.66 (0.55, 5.01)	1.29 (0.36, 4.66)
No	No	1.00 (reference)	1.00 (reference)	1.00 (reference)	1.00 (reference)
	Yes	**1.48 (1.30, 1.68)**	**1.18 (1.02, 1.35)**	**2.35 (1.81, 3.05)**	**1.71 (1.30, 2.26)**
P for multiplicative interaction		0.084	0.30	0.55	0.63
**Marital status**					
Single/divorced/widow	No	1.00 (reference)	1.00 (reference)	1.00 (reference)	1.00 (reference)
	Yes	**1.78 (1.20, 2.65)**	1.53 (0.96, 2.43)	1.06 (0.58, 1.97)	0.82 (0.40, 1.72)
Married	No	1.00 (reference)	1.00 (reference)	1.00 (reference)	1.00 (reference)
	Yes	**1.33 (1.17, 1.52)**	1.10 (0.96, 1.26)	**2.68 (2.03, 3.54)**	**1.91 (1.42, 2.56)**
P for multiplicative interaction		0.18	0.30	**0.007**	**0.026**
**Education level**					
Junior middle school or below	No	1.00 (reference)	1.00 (reference)	1.00 (reference)	1.00 (reference)
	Yes	**1.51 (1.26, 1.82)**	**1.22 (1.00, 1.49)**	**2.43 (1.75, 3.37)**	**1.76 (1.24, 2.49)**
Senior middle school	No	1.00 (reference)	1.00 (reference)	1.00 (reference)	1.00 (reference)
	Yes	1.25 (0.99, 1.47)	1.10 (0.86, 1.41)	**2.23 (1.31, 3.80)**	1.60 (0.90, 2.83)
College or above	No	1.00 (reference)	1.00 (reference)	1.00 (reference)	1.00 (reference)
	Yes	**1.66 (1.33, 2.08)**	1.12 (0.86, 1.46)	1.78 (0.94, 3.39)	1.39 (0.69, 2.80)
P for multiplicative interaction		0.19	0.48	0.70	0.83
**Occupation type**					
Administrators/Professionals	No	1.00 (reference)	1.00 (reference)	1.00 (reference)	1.00 (reference)
	Yes	**1.65 (1.24, 2.20)**	1.10 (0.78, 1.56)	**2.44 (1.07, 5.60)**	1.90 (0.75, 4.81)
Workers	No	1.00 (reference)	1.00 (reference)	1.00 (reference)	1.00 (reference)
	Yes	**1.45 (1.17, 1.79)**	**1.29 (1.02, 1.63)**	**2.69 (1.86, 3.89)**	**1.64 (1.10, 2.45)**
Business services personnel	No	1.00 (reference)	1.00 (reference)	1.00 (reference)	1.00 (reference)
	Yes	1.14 (0.84, 1.56)	0.85 (0.60, 1.21)	2.05 (0.94, 4.47)	1.66 (0.70, 3.92)
Housewife/unemployed women	No	1.00 (reference)	1.00 (reference)	1.00 (reference)	1.00 (reference)
	Yes	**1.60 (1.20, 2.13)**	**1.38 (1.01, 1.89)**	1.76 (0.92, 3.37)	1.26 (0.62, 2.56)
Others	No	1.00 (reference)	1.00 (reference)	1.00 (reference)	1.00 (reference)
	Yes	**1.57 (1.16, 2.12)**	1.23 (0.87, 1.74)	**2.59 (1.37, 4.89)**	1.87 (0.92, 3.76)
P for multiplicative interaction		0.45	0.22	0.83	0.96
**Monthly income (CNY)**					
< 5,000	No	1.00 (reference)	1.00 (reference)	1.00 (reference)	1.00 (reference)
	Yes	**1.55 (1.32, 1.82)**	**1.22 (1.03, 1.46)**	**2.44 (1.75, 3.40)**	**1.54 (1.08, 2.21)**
5,000–7999	No	1.00 (reference)	1.00 (reference)	1.00 (reference)	1.00 (reference)
	Yes	**1.33 (1.07, 1.66)**	1.01 (0.79, 1.30)	**2.31 (1.49, 3.57)**	**2.07 (1.29, 3.34)**
≥ 8000	No	1.00 (reference)	1.00 (reference)	1.00 (reference)	1.00 (reference)
	Yes	**1.39 (1.01, 1.92)**	1.03 (0.71, 1.50)	1.92 (0.78, 4.69)	1.25 (0.43, 3.61)
P for multiplicative interaction		0.54	0.55	0.88	0.73
**Health insurance**					
No	No	1.00 (reference)	1.00 (reference)	1.00 (reference)	1.00 (reference)
	Yes	0.75 (0.49, 1.14)	0.63 (0.40, 1.00)	1.31 (0.73, 2.36)	0.74 (0.37, 1.46)
Yes	No	1.00 (reference)	1.00 (reference)	1.00 (reference)	1.00 (reference)
	Yes	**1.48 (1.30, 1.68)**	**1.21 (1.05, 1.39)**	**2.56 (1.93, 3.39)**	**1.97 (1.47, 2.64)**
P for multiplicative interaction		**0.002**	** < 0.001**	**0.044**	**0.005**
**Age at sexual debut (year)**					
< 18	No	1.00 (reference)	1.00 (reference)	1.00 (reference)	1.00 (reference)
	Yes	1.05 (0.72, 1.53)	0.81 (0.53, 1.26)	1.30 (0.66, 2.57)	0.93 (0.42, 2.07)
18–24	No	1.00 (reference)	1.00 (reference)	1.00 (reference)	1.00 (reference)
	Yes	**1.37 (1.19, 1.58)**	1.11 (0.95, 1.30)	**2.55 (1.88, 3.46)**	**1.82 (1.32, 2.51)**
≥ 25	No	1.00 (reference)	1.00 (reference)	1.00 (reference)	1.00 (reference)
	Yes	**2.25 (1.69, 3.00)**	**1.59 (1.16, 2.18)**	**2.89 (1.54, 5.41)**	1.89 (0.96, 3.74)
P for multiplicative interaction		**0.002**	**0.009**	0.17	0.19
**The number of lifetime sexual partners**					
1	No	1.00 (reference)	1.00 (reference)	1.00 (reference)	1.00 (reference)
	Yes	**1.53 (1.32, 1.76)**	**1.17 (1.01, 1.37)**	**2.89 (2.16, 3.85)**	**1.93 (1.42, 2.63)**
2	No	1.00 (reference)	1.00 (reference)	1.00 (reference)	1.00 (reference)
	Yes	**1.50 (1.12, 2.00)**	1.16 (0.84, 1.61)	1.52 (0.80, 2.91)	1.05 (0.52, 2.13)
≥ 3	No	1.00 (reference)	1.00 (reference)	1.00 (reference)	1.00 (reference)
	Yes	1.23 (0.85, 1.79)	1.04 (0.67, 1.60)	1.12 (0.43, 2.88)	0.86 (0.29, 2.54)
P for multiplicative interaction		0.58	0.35	0.051	0.061
**Consistent condom use during sexual intercourse**					
No	No	1.00 (reference)	1.00 (reference)	1.00 (reference)	1.00 (reference)
	Yes	**1.39 (1.22, 1.59)**	1.08 (0.93, 1.24)	**2.50 (1.91, 3.27)**	**1.71 (1.28, 2.28)**
Yes	No	1.00 (reference)	1.00 (reference)	1.00 (reference)	1.00 (reference)
	Yes	**1.94 (1.46, 2.57)**	**1.55 (1.11, 2.16)**	1.68 (0.79, 3.61)	1.20 (0.52, 2.76)
P for multiplicative interaction		**0.038**	**0.042**	0.34	0.59
**Parity**					
0	No	1.00 (reference)	1.00 (reference)	1.00 (reference)	1.00 (reference)
	Yes	**1.61 (1.09, 2.38)**	1.51 (0.97, 2.35)	1.64 (0.85, 3.17)	1.52 (0.73, 3.19)
1	No	1.00 (reference)	1.00 (reference)	1.00 (reference)	1.00 (reference)
	Yes	1.13 (0.91, 1.40)	0.94 (0.74, 1.19)	**2.39 (1.51, 3.77)**	1.57 (0.96, 2.58)
2	No	1.00 (reference)	1.00 (reference)	1.00 (reference)	1.00 (reference)
	Yes	**1.47 (1.22, 1.76)**	1.20 (0.98, 1.46)	**2.28 (1.56, 3.31)**	**1.55 (1.04, 2.32)**
≥ 3	No	1.00 (reference)	1.00 (reference)	1.00 (reference)	1.00 (reference)
	Yes	1.39 (0.96, 2.01)	1.28 (0.84, 1.94)	**5.15 (2.04, 13.02)**	**3.33 (1.22, 9.10)**
P for multiplicative interaction		0.24	0.25	0.27	0.33
**HPV-related knowledge**					
Low level (< 6)	No	1.00 (reference)	1.00 (reference)	1.00 (reference)	1.00 (reference)
	Yes	**1.56 (1.32, 1.83)**	**1.39 (1.17, 1.65)**	**2.00 (1.52, 2.62)**	**1.61 (1.21, 2.14)**
High level (≥ 6)	No	1.00 (reference)	1.00 (reference)	1.00 (reference)	1.00 (reference)
	Yes	1.16 (0.96, 1.40)	0.94 (0.76, 1.16)	**2.26 (1.03, 4.94)**	1.80 (0.78, 4.16)
P for multiplicative interaction		**0.019**	**0.004**	0.77	0.42

^a^Model 1: unadjusted

^b^Model 3: adjusted for age, ethnicity, local household registration, marital status, education level, occupation type, monthly income, health insurance, age at sexual debut, the number of lifetime sexual partners, consistent condom use during sexual intercourse, parity, and HPV-related knowledge (the corresponding factor was not included when being regarded as a modified factor)

Similarly, signing up with family doctors was positively associated with future screening willingness in the adjusted model (OR: 1.68, 95%CI: 1.29–2.20) (Fig. 2). This association was more pronounced among women who got married (OR: 1.91, 95%CI: 1.42–2.56) and had health insurance (OR: 1.97, 95%CI: 1.47–2.64). Multiplicative interactions of the signing status with marital status and health insurance were detected (P for multiplicative interactions: 0.026 and 0.005, respectively) (Table 4).

## Discussion

Identifying potential facilitators and enhancing participation in cervical cancer screening greatly help to achieve cervical cancer elimination targets. To our knowledge, this study is the first investigation to link FDCS with cervical cancer screening behaviors. Notably, women signing up with family doctors tended to have higher past screening participation and future screening willingness, suggesting the application value of promoting cervical cancer screening through FDCS.

FDCS is a core part of the primary healthcare system opening to the general public. The signing rate of FDCS in the current study was 54.3%, which was similar to the findings in Guangdong (54.7%) and Zhejiang province (50.43%) [14, 15], but much higher than that from a nationwide survey in mainland China (approximately 6.0% in the female population) [16]. Researchers also found lower signing rates among people over 60 years old (28.2%) and with chronic diseases (29.3%) in rural China [17, 18]. Here, the socioeconomic and regional differences may provide a potential explanation for these inconsistencies to some extent. As prior studies suggested, the signing rate may differ by the survey year, region, and household residence status [19]. Moreover, people’s signing behavior may be affected by the awareness of FDCS as well as sociodemographic characteristics (e.g. age, education, marital status, and household registration) [20]. The Chinese government aims to expand FDCS to cover the entire population by 2020 [15], however, the present signing rate in Shenzhen city is still far from this goal. In recent years, the reform of the primary health-care system has persisted by establishing networks of integrated management, shared responsibilities, and common interests between community health service centers and hospitals in Shenzhen [21], which gradually orients residents toward primary healthcare. With the improvement of primary healthcare system capacity, health education should be conducted to increase public awareness and utilization of FDCS.

As a nationally representative survey reported, only one in five Chinese females aged ≥ 21 years have screened for cervical cancer, with geographical and socioeconomic variations [22]. It’s urgently needed to accelerate the increase in the awareness and accessibility of cervical cancer screening in China. Here, our survey showed that women had better HPV-related knowledge and cervical cancer screening behaviors in Shenzhen city. In the current study, women demonstrated a lower proportion (57.1%) of low HPV-related knowledge levels when compared to our previous survey in 2015 (67.6%) regardless of different question items [23]. Both the past screening rate (44.0%) and future screening willingness (93.6%) were also higher than the data from our previous surveys in 2014 (35.1% and 82.8%, respectively) [24]. These improved indicators may be owing to a policy that cervical cancer screening has been included in the basic public health services of Shenzhen city since 2017, accompanied by an expansion in the coverage of cervical cancer prevention related work (e.g. health education, technical training, and organized screening). However, these achievements still have a certain distance to the WHO cervical cancer elimination target by 2030 (70% coverage of twice-lifetime screening) [25]. Furthermore, there was a notable gap between past screening participation and future screening willingness in our survey, implying the fact that more than half of those willing to be screened remained unscreened. Hence, future health interventions should be placed on the shift from screening willingness to screening practice.

It’s proved that primary healthcare settings have strengths in cancer prevention because the physicians contribute to information provision as well as the promotion of screening uptake and informed choice [26]. In this study, our findings shed light on the positive impacts of family doctors to guide cervical cancer prevention. Women signing up with family doctors performed better in HPV-related knowledge, past screening participation, and future screening willingness than their non-signing counterparts. These results were similar to those of studies in other countries that general practitioners helped to increase participation in cervical cancer screening [12, 13, 27]. Researchers further detected that high awareness of health promotion among general practitioners achieved high rates of cervical cancer screening in a multinational modeling study [28]. Moreover, educational intervention for general practitioners about the importance of cervical cancer screening was effective in increasing the uptake of screening among immigrants [29]. As the core members of the family doctor team, on one hand, general practitioners may contribute to providing screening recommendations that affect patients’ healthcare decisions, which has been identified as a facilitator of cervical cancer screening behavior [7, 30, 31]. In this context, women who know little about cervical cancer may receive more benefits from family doctors. As our findings suggested, the association between signing up with family doctors and past screening participation was confined to women with a low level of HPV-related knowledge. On the other hand, the facilitating effects may be also explained by a specific service advantage that some members (general practitioners or specialized doctors) of the family doctor team usually undertake gynecological examination and cervical sampling procedures. A cross-sectional survey in Poland found that nearly two-thirds of patients were willing to undergo cervical cancer screening by their family doctors [32]. Therefore, FDCS may attract signing women to engage in screening behaviors as a result of operational accessibility. Additionally, we noticed that the association between signing up with family doctors and cervical cancer screening behaviors tended to be more robust among women who were married, had health insurance, or behaved more discreetly during sexual behavior. These women may have better adherence to family doctors owing to more concerns for healthcare issues. Both the signing status and cervical cancer screening behaviors could be affected by marital status, health insurance, and sexual behaviors [15, 33, 34], therefore, further investigations should be conducted to verify these potential relationships.

There were some limitations in this study. Firstly, we could not infer causal relationships between signing up with family doctors and cervical cancer screening behaviors due to the cross-sectional design. Secondly, as the survey sampling site were restricted to be community health service centers in the Baoan district of Shenzhen city, the implications of our findings might not be able to generalize to the whole population. Thirdly, signing women were not required to report the demographic characteristics of their family doctors (e.g. age, gender, major, and working years) and the details of daily health guidance in our survey, which may restrict to investigating the role of family doctors in facilitating cervical cancer screening behaviors. Thus, more longitudinal studies with a comprehensive evaluation of FDCS are needed.

## Conclusions

Family doctors play a gatekeeper role in the primary healthcare system in China. The self-reported rates of signing up with family doctors and cervical cancer screening participation were acceptable among women in Shenzhen city. Notably, signing up with family doctors was associated with cervical cancer screening behaviors, including past screening participation and future screening willingness. Expanding public awareness of cervical cancer prevention and FDCS may be a feasible way to achieve the goal of cervical cancer screening coverage.

## Data Availability

The raw data supporting the conclusions of this article will be made available by the corresponding author, upon reasonable request.

## Abbreviations

HPV: Human papillomavirus
FDCS: Family doctor contract services
OR: Odds ratio
